# Self-Explainable Graph Neural Network for Alzheimer Disease and Related Dementias Risk Prediction: Algorithm Development and Validation Study

**DOI:** 10.2196/54748

**Published:** 2024-07-08

**Authors:** Xinyue Hu, Zenan Sun, Yi Nian, Yichen Wang, Yifang Dang, Fang Li, Jingna Feng, Evan Yu, Cui Tao

**Affiliations:** 1 Department of Artificial Intelligence and Informatics Mayo Clinic Jacksonville, FL United States; 2 McWilliams School of Biomedical Informatics The University of Texas Health Science Center at Houston Houston, TX United States; 3 Division of Hospital Medicine at Perelman School of Medicine The University of Pennsylvania Philadelphia, PA United States

**Keywords:** Alzheimer disease and related dementias, risk prediction, graph neural network, relation importance, machine learning

## Abstract

**Background:**

Alzheimer disease and related dementias (ADRD) rank as the sixth leading cause of death in the United States, underlining the importance of accurate ADRD risk prediction. While recent advancements in ADRD risk prediction have primarily relied on imaging analysis, not all patients undergo medical imaging before an ADRD diagnosis. Merging machine learning with claims data can reveal additional risk factors and uncover interconnections among diverse medical codes.

**Objective:**

The study aims to use graph neural networks (GNNs) with claim data for ADRD risk prediction. Addressing the lack of human-interpretable reasons behind these predictions, we introduce an innovative, self-explainable method to evaluate relationship importance and its influence on ADRD risk prediction.

**Methods:**

We used a variationally regularized encoder-decoder GNN (variational GNN [VGNN]) integrated with our proposed relation importance method for estimating ADRD likelihood. This self-explainable method can provide a feature-important explanation in the context of ADRD risk prediction, leveraging relational information within a graph. Three scenarios with 1-year, 2-year, and 3-year prediction windows were created to assess the model’s efficiency, respectively. Random forest (RF) and light gradient boost machine (LGBM) were used as baselines. By using this method, we further clarify the key relationships for ADRD risk prediction.

**Results:**

In scenario 1, the VGNN model showed area under the receiver operating characteristic (AUROC) scores of 0.7272 and 0.7480 for the small subset and the matched cohort data set. It outperforms RF and LGBM by 10.6% and 9.1%, respectively, on average. In scenario 2, it achieved AUROC scores of 0.7125 and 0.7281, surpassing the other models by 10.5% and 8.9%, respectively. Similarly, in scenario 3, AUROC scores of 0.7001 and 0.7187 were obtained, exceeding 10.1% and 8.5% than the baseline models, respectively. These results clearly demonstrate the significant superiority of the graph-based approach over the tree-based models (RF and LGBM) in predicting ADRD. Furthermore, the integration of the VGNN model and our relation importance interpretation could provide valuable insight into paired factors that may contribute to or delay ADRD progression.

**Conclusions:**

Using our innovative self-explainable method with claims data enhances ADRD risk prediction and provides insights into the impact of interconnected medical code relationships. This methodology not only enables ADRD risk modeling but also shows potential for other image analysis predictions using claims data.

## Introduction

### Background

Alzheimer disease and related dementias (ADRD) currently rank as the sixth leading cause of death in the United States [1]. Currently, 47 million people live with ADRD globally [2]. By the year 2050, the prevalence of dementia is expected to triple worldwide [3]. These alarming statistics emphasize the pressing need for accurately predicting ADRD risk, which holds immense significance for several reasons. First, it enables early detection and diagnosis, which can facilitate timely interventions and treatment plans that have the potential to slow down disease progression, improve patient outcomes, and enhance the quality of life for individuals affected by ADRD. Second, it also plays a crucial role in advancing research and drug development. It provides valuable insights into disease progression, risk factors, and potential therapeutic targets. By identifying individuals at high risk of developing ADRD, researchers can conduct targeted studies and clinical trials and explore preventive measures to mitigate the impact of this debilitating disease. Third, early prediction and intervention may help reduce health care costs associated with ADRD. By identifying individuals at risk and providing appropriate care, the burden on the health care system can be lessened. Nevertheless, predicting ADRD risks is an intricate task due to its nature as a long-term chronic disease with multifaceted underlying causes.

In the context of ADRD risk prediction, the conventional approach predominantly involves using machine learning (ML) models with medical imaging data as primary resources to achieve commendable success [4-6]. However, it is important to acknowledge that not all patients undergo routine clinical imaging tests during their regular visits, rendering medical imaging data less accessible for certain individuals. In contrast, claims data provide a more readily available data source for the ML predictors. Hence, the development of a valuable and easily trainable risk prediction tool necessitates the use of existing claims data as the primary input for prediction. This approach not only enhances the model’s generalizability but also facilitates its adaptation to other diverse data sources.

In recent years, the emergence of graph-structured data has received significant interest within the realm of deep learning [7-11]. Graphs are composed of nodes and relationships, resulting in the representation and analysis of intricate connections and patterns within the data they encapsulate. They also offer a unique combination of topological structure and individual features, which enables a rich source of information [12,13]. To analyze and model the complex relations of interconnected graph data, graph neural networks (GNNs) have emerged as a powerful tool [14]. Unlike traditional ML models that operate on fixed-dimensional inputs, GNNs operate directly on the graph structure, which allows them to learn the representation of individuals, attributes, and relationships. In the biomedical domain, GNNs have been used for tasks such as protein function prediction, drug discovery, disease classification, and personalized medicine [15-20]. Li et al [21] proposed a multi-channel GNN for predicting drug-target interactions that combines a multi-channel graph convolutional network and graph attention network (GAT). This framework uses a topology graph for contextual representation, a feature graph for semantic representation, and a common representation of drug and protein pairs. It has demonstrated remarkable accuracy in identifying drug-target interactions, achieving an impressive area under the receiver operating characteristic (AUROC) score of 0.9665. Wang et al [22] introduced a deep learning framework, Deep Learning for Drug-Drug Synergy prediction (DeepDDS), for predicting drug-drug interactions for anticancer treatments. DeepDDS uses gene expression data from the cancer cell line and the molecular graph of the drugs as input. It leverages GAT and graph convolution transformers (GCTs) to accurately predict the synergistic effect between drug combinations. DeepDDS has achieved an AUROC score of 0.67 on an independent test set. In the task of ADRD prediction, GCT obtained an area under the precision-recall curve of 0.34 on the inpatient and outpatient electronic health record (EHR) data from NYU Langone Health (briefly called AD-EHR) [23]. Klepl et al [24] integrated functional connectivity methods with GNNs to evaluate ADRD prediction performance using electroencephalography brain data. They showed that the GNN-based approach outperformed convolutional neural network and support vector machine models and obtained an AUROC of 0.984 [24]. Zhu and Razavian [23] presented variational GNN (VGNN), a variationally regularized encoder-decoder GNN, designed specifically for EHRs. This framework showed robustness in learning graph structures by applying regularization techniques to node representations. VGNN was used for ADRD risk prediction, and it attained an area under the precision-recall curve of 0.46 when using AD-EHR.

The abovementioned GNN models [23,24] have demonstrated the potential to uncover hidden patterns, reveal biological insights, and facilitate advancements in ADRD prediction. However, because the GNN architecture is a black-box model, the absence of interpretability is harmful to both users and society [25], especially in critical applications where decisions need to be explained or understood. Even though some advanced models such as GAT, GCT, and VGNN have the ability to explain the importance of individual nodes by using attention mechanisms, they still face a limitation in their interpretability concerning the significance of underlying relationships in the prediction process. As a consequence, there is a pressing demand for research and development efforts to enhance GNNs and elucidate the influence of relationship importance in achieving more precise ADRD predictions. By addressing this interpretability issue, GNNs can become more valuable tools in advancing our understanding of ADRD and contributing to improved patient care and treatment strategies.

### Objective

The first focus of this study lies in the domain of risk prediction for ADRD. In this particular context, our investigation aims to use claims data as the sole input for our GNN-based predictive model for accurate ADRD risk prediction. We enhance the predictive power of our model by incorporating advanced GNN models into a framework that enables us to effectively capture the intricate relationships and dependencies inherent in the claims data.

Second, we introduced a novel method to assess the importance of relationships within the patients’ individual medical record graphs and their influence on ADRD risk prediction. Generally, an additional graph explanation technique, such as GNNExplainer [26], is used as a post hoc method to interpret the predictions made by the GNNs. However, our proposed relation importance method enables an “in-process” explanation approach that leverages the relation weights from each patient’s individual graph. This method facilitates the interpretation of the GNN’s predictions during the graph generation process itself. Besides that, our method aims to adequately calibrate the importance of each relationship within the graph, reflecting their true impact on prediction. Since, typically, when a relation connects to nodes that are highly prevalent in the graph, there is a risk of misdefining its significance. The frequent occurrence of these nodes can distort the perception of the relationship’s importance, potentially leading to erroneous interpretations or biased conclusions. This bias can result in a skewed importance assigned to relationships, and hence potentially affecting the accuracy of ADRD risk prediction. By considering the patient groups with and without ADRD, our approach helps to mitigate the potential bias resulting from node frequency, enabling a more comprehensive and reliable interpretability of relation importance for ADRD risk prediction.

## Methods

### Cohort Description

We used deidentified administrative health claims data from Optum’s Clinformatics Data Mart, spanning from 2007 to 2020. This data set comprises over 68 million patient-level enrollment records submitted by various health care providers, pharmacies, and other health care service organizations for reimbursement purposes. It is accessible for researchers through a subscription provided by the University of Texas Health Science Center (UTHealth) [27].

**Figure 1:**
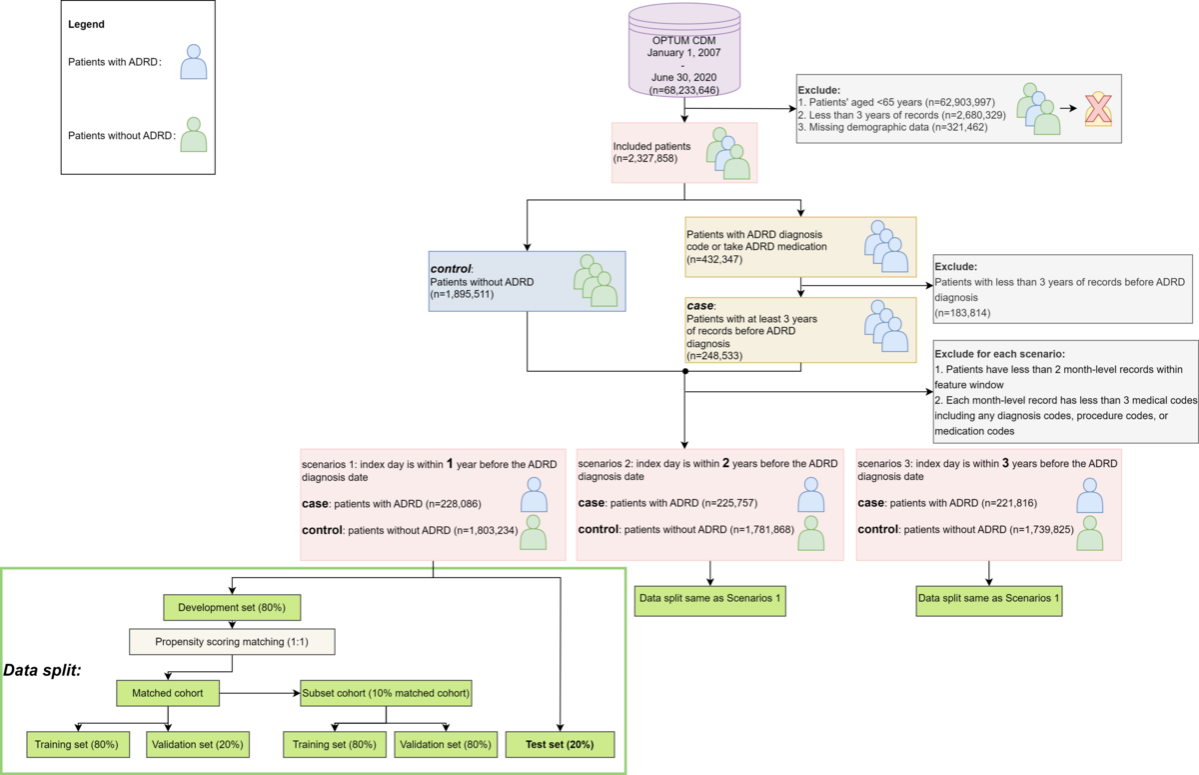
Overview of cohort selection for 3 scenarios. ADRD: Alzheimer disease and related dementias.

Several criteria were applied to construct the study cohort, as illustrated in Figure 1. Considering that ADRD primarily affects older individuals and is a chronic condition, we initially filtered out patients (n=62,903,997) who were younger than 65 years. To ensure a sufficient data history for tracking their medical conditions, patients (n=2,680,329) with a time span of less than 3 years between their initial and final medical records were excluded. Patients (n=321,462) who lacked demographic information were also excluded from the study. To further establish the ADRD cohort, we used the definition outlined by Kim et al [28]. Patients were classified as having ADRD if they presented specific diagnosis codes or were prescribed relevant medications. The specified diagnosis codes are Alzheimer dementia (331.0*/G30.*), vascular dementia (290.4*/F01.*), frontotemporal dementia (331.1*/G31.0*), lewy body dementia (331.82*/G31.83), senile dementia (290.0*), presenile dementia (290.1*), other specified senile psychotic (290.8*), and unspecified senile psychotic condition (290.9*), and the medication includes aricept, donepezil, razadyne, reminyl, galantamine, exelon, rivastigmine, namenda, memantine, acetylcholine, and memantine. Based on the criteria mentioned above, the resulting cohort included 432,374 patients with ADRD and 1,895,511 patients without ADRD.

### Data Preprocessing

**Figure 2:**
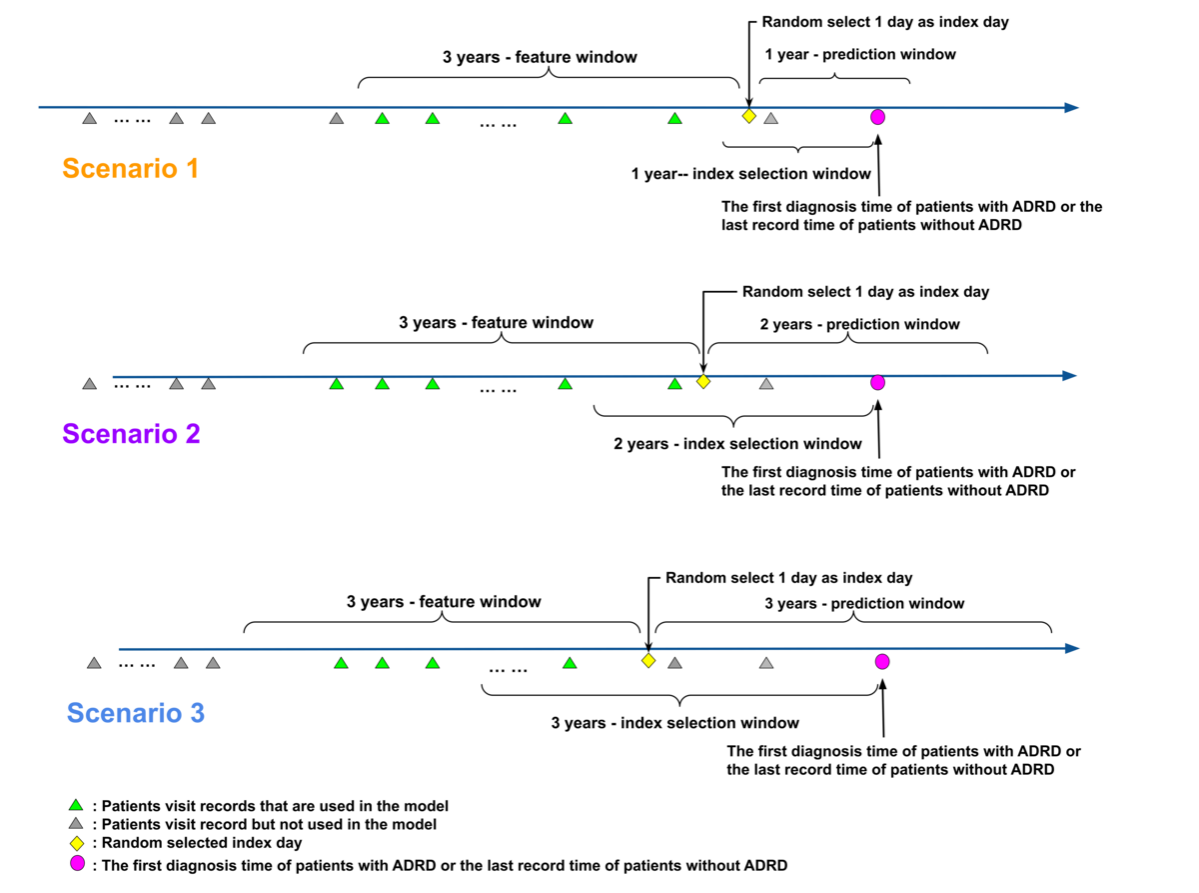
The definition of 3 scenarios. We established a time frame that includes an index selection window, a feature window, and a prediction window. The index selection window spanned a specific period before the initial diagnosis of patients with Alzheimer disease and related dementias (ADRD) or the last record for patients without ADRD. We randomly selected a day within the index selection window as the index day to simulate real-world visiting settings. The period up to 3 years before this index day was considered the feature window for training the model, while the period after the index day was used as the prediction window. We used 1 year, 2 years, and 3 years as the lengths of the index selection window and corresponding prediction window, respectively, to predict ADRD diagnosis dynamically.

In this study, we used a partitioning approach to categorize each patient’s records into 3 time windows, such as an index selection window, a feature window, and a prediction window (shown in Figure 2). First, we designated a specific period before the initial diagnosis of patients with ADRD or the last record for patients without ADRD as the index selection window. In the real world, patients may seek consultations for their health conditions at any time. To simulate this visiting setting, we randomly select the index day within each patient’s index selection window instead of using a fixed day. The 3-year period before the index day serves as the feature window for model training purposes, while a certain period after the index day is defined as the “prediction window” for ADRD risk prediction. Additionally, we designed 3 scenarios with index selection windows and prediction windows of 1, 2, and 3 years in length, respectively. By using this partitioning approach, we can comprehensively evaluate our model’s predictive accuracy in dynamically predicting ADRD diagnoses. It should be noted that researchers can easily adjust the lengths of these windows to align with their specific requirements and objectives.

There are also other inclusion criteria that were applied to ensure the quality of the data and the fairness of the cohort. Specifically, within the feature window, it was required that each patient have a minimum of 2 month-level records. Furthermore, within the records in the same month, a minimum of 3 medical codes (eg, diagnosis codes, procedure codes, and medication codes) needed to be present. After applying these criteria, the resulting cohort for each scenario is presented in Figure 1. In scenario 1, the cohort consisted of a total of 2,031,320 patients, comprising 228,086 patients with ADRD and 1,803,234 patients without ADRD. For scenario 2, the cohort comprised 2,007,625 patients, including 225,757 patients with ADRD and 1,781,868 patients without ADRD. Finally, in scenario 3, the cohort encompassed 1,961,641 patients, with 221,816 patients with ADRD and 1,739,825 patients without ADRD. These cohorts provide a robust foundation for further analysis and investigation in this study.

The data used in all cohorts included claims data consisting of diagnoses encoded with both International Classification of Diseases, 9th revision (ICD-9) codes and 10th version (ICD-10) codes, the National Drug Code for pharmacy claims, current procedural terminology, and Healthcare Common Procedure Coding System codes for procedures. The inclusion of both ICD-10 and ICD-9 codes was necessary as the study period spanned the transition from ICD-9 to ICD-10 coding systems. All these different types of medical codes have been converted to a higher-level categorization scheme to achieve feature reduction, uniformity, and compatibility within the study analysis. The ICD-9 and ICD-10 codes and the current procedural terminology and Healthcare Common Procedure Coding System codes are converted to clinical classification software, which is a tool for clustering patient diagnoses and procedures into a manageable number of clinically meaningful categories developed at the Agency for Healthcare Research and Quality (formerly known as the Agency for Health Care Policy) [29]. Similarly, we are using the Pharmacologic-Therapeutic Classification System from the American Hospital Formulary Service to represent and group the drug National Drug Code in the data set [30]. It is a method of grouping drugs with similar pharmacologic, therapeutic, and chemical characteristics in a 4-tier hierarchy associated with a numeric code consisting of 2 to 8 digits. By following the conversion of these codes, the number of features was reduced from tens of thousands to hundreds. This reduction not only helps address the issue of sparsity in the model input but also improves its overall efficiency.

### Modeling

We used the VGNN in combination with patients’ diagnosis, procedure, and medication codes to estimate the likelihood of patients having ADRD within a designated prediction window. VGNN consists of 4 modules, such as the encoder graph, variational regularization, decoder graph, and fully connected layer. In the encoder graph module, VGNN takes 3 types of patients’ medical codes from the feature window as input and constructs a fully connected graph comprising medical codes for each patient. The representation of each node is iteratively updated through multiple graph attention layers. To address the challenges of generating node embeddings within clusters and achieving balanced attention weights, VGNN incorporates a variational regularization layer. This layer helps prevent model collapse and maintains the model’s expressive capacity. The decoder graph module uses the node representations generated by the encoder graph and the variational regularization layer to compute the weighted relations between each node. These weighted relationships effectively capture the relationships among different medical codes. Finally, a linear feed-forward layer is used to calculate the probability and produce the binary classification for identifying an individual with ADRD.

**Figure 3:**
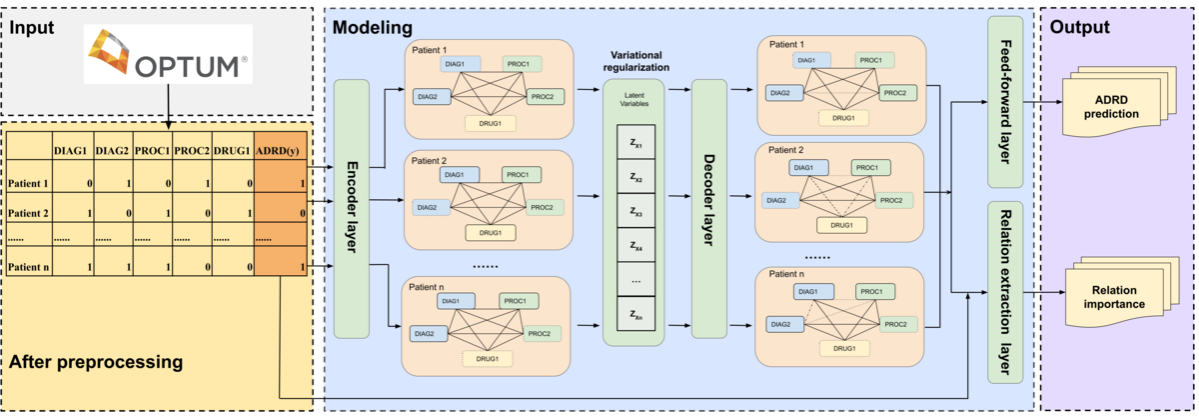
The workflow of sour study pipeline, including data preprocessing, graph modeling, and final output. The DIAG, PROC, and DRUG represent 3 types of medical codes: diagnosis, procedure, and medication, respectively. We used the variationally regularized encoder-decoder graph neural network (VGNN) to predict the likelihood of Alzheimer disease and related dementias (ADRD) using patients’ medical records sourced from Optum Clinformatics. The data were input into the encoder layer of VGNN, generating a fully connected graph specific to each patient. The variational regularization layer was then applied to prevent issues like mode collapse and maintain the model’s capacity to represent information effectively. Additionally, the decoder graph module used node representations to compute weighted relations between nodes, which effectively captured relationships among different medical codes. Finally, a linear feed-forward layer was used to calculate probabilities and perform binary classification.

We initiated the modeling process by reserving 20% of patients from the entire data set for testing purposes. Given that ADRD is more prevalent in the older population [1] and our data set exhibits a high imbalance, we used the propensity score matching method based on age and gender to mitigate potential biases associated with these factors. This matching process ensured that our model’s input cohort consisted of individuals with similar age and gender distributions, reducing the potential confounding effects associated with these variables. As a result, we created a balanced cohort with a one-to-one ratio of control and case groups from the remaining 80% of the entire data set. This downsampling approach is a popular method in clinical research to create a balanced covariate distribution between treated and untreated groups, which could help significantly improve the model’s ability to handle imbalanced data [31]. We named it the matched cohort and used it for the purposes of model development and validation. Additionally, we generated a smaller subset named the subset cohort, which is 10% of the matched cohort. This action allows us to evaluate the model’s performance on a smaller-scale data set effectively. In order to assess the efficacy of our approach, we built models for 3 different scenarios. Moreover, we used random forest (RF) and light gradient boost machine (LGBM) as baseline models and compared their performance with that of VGNN. The overall workflow of our model pipeline is shown in Figure 3.

### Relation Importance

After the completion of model training, we then used the trained model to build the interconnected medical record graph for each individual patient. In order to evaluate the significance of various relationships in ADRD prediction, we extracted adjacency matrices 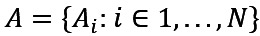. from the medical graphs of N patients in the training set of the matched cohort. The values within these adjacency matrices serve as indicators of the relational importance associated with predicting ADRD. Given that the generated graphs are directional, the adjacency matrices *A* are not symmetric. Therefore, we took an additional step to mitigate the influence of directionality by computing the average of the original adjacency matrix and its transposed matrix. Then, the updated adjacency matrix is:



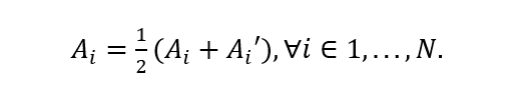



This adjacency matrix enables us to gain insights into the intricate relationships between medical codes and their predictive power for ADRD.

However, it is crucial to consider that medical codes with higher frequencies may have received relatively larger weights compared to others, potentially introducing bias in the analysis. Given that *A*^+^ are the adjacency matrices of patients with ADRD case group and *A*^–^are the adjacency matrices of ADRD patient control group, we calculated the mean adjacency matrix of these 2 patient groups as:



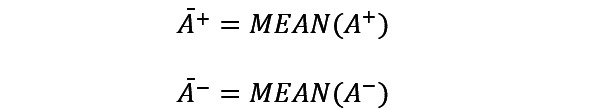



By subtracting the negative mean adjacency matrix from the positive mean adjacency matrix, we eventually obtained a mean weight-difference matrix:



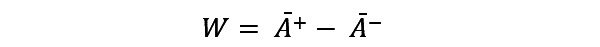



This mean weight-difference matrix *W* captured the relative significance of the medical code weights. A higher positive value inside *W* indicates a greater importance in predicting ADRD, while a lower negative value suggests a reduced likelihood of ADRD occurrence. A value of 0 in *W* means that the relationship does not affect a patient with ADRD.

### Ethical Considerations

The approval for the use of data in this study was obtained from the UTHealth Committee for the Protection of Human Subjects, under protocol HSC-SBMI-21-0965, with a waiver of consent granted.

## Results

### Hyperparameter Setting

We trained the VGNN model with the following hyperparameters: a learning rate of 0.0001, a batch size of 128, and a dropout rate of 0.1. We used the Adam optimizer for gradient descent and trained the model for 200 epochs. The model consisted of 2 graph layers and 1 attention head. To balance the binary cross-entropy and Kullback-Leibler divergence losses, a parameter value of 0.002 was used. Additionally, edge information was extracted after the attention layer to facilitate future calculations of relational importance. Additionally, we used the grid search method to tune the RF and LGBM baseline models. The hyperparameters for RF and LGBM are n_estimators=100, min_samples_split=2, and min_samples_leaf=1, and n_estimators=300, boosting_type=“gbdt,” num_leaves=31, and learning_rate=0.1, respectively.

### Performance Evaluation

We used AUROC as a measurement to evaluate the performance of each model. As shown in Table 1, the VGNN model achieved AUROC scores of 0.7272 and 0.7480 for the subset cohort and the matched cohort, respectively, in scenario 1. It outperformed the RF and LGBM models by an average of 10.6% and 9.1% across the 2 data sets. For scenario 2, the VGNN model obtained AUROC scores of 0.7125 and 0.7281 for the subset cohort and matched cohort, respectively. It exhibited superior performance compared to the other 2 models by an average of 10.5% and 8.9% across the 2 data sets. Finally, in scenario 3, the VGNN model achieved AUROC scores of 0.7001 and 0.7187, which were surpassed by an average of 10.1% and 8.5% across the 2 data sets. The results clearly demonstrate that the GNN approach (VGNN) outperforms the tree-based models (RF and LGBM) significantly in predicting ADRD. The bar chart for the performance comparison can be found in Multimedia Appendix 1.

Furthermore, we identified the 5 most important relationships for both positive and negative predictions of ADRD in Table 2. Among the top 5 negative highest-weighted relationships, “neoplasms of unspecified nature or uncertain behavior” exhibits its influence across all relations within scenario 1, “consultation, evaluation, and preventative care” makes a total of 4 appearances within scenario 2, while “quinolone antibiotics” spans all relations in scenario 3. Within the set of the top 5 positive highest-weighted relationships, both “routine chest x-ray” and “electrocardiogram” appear 3 times each in scenario 1, “substance-related disorders” contributes to 4 relationships in scenario 2, and “substance-related disorders” emerges as the most frequently occurring medical code in scenario 3.

**Table 1:** The model performance (area under the receiver operating characteristic curve scores) for Alzheimer disease and related dementias risk prediction.

Scores			Random forest		Light gradient boost machine		Variational graph neural network
**Matched cohort**							
	Scenario 1	0.6710		0.6809		0.7480	
	Scenario 2	0.6565		0.6658		0.7281	
	Scenario 3	0.6468		0.6589		0.7187	
**Subset cohort**							
	Scenario 1	0.6629		0.6720		0.7272	
	Scenario 2	0.6474		0.6570		0.7125	
	Scenario 3	0.6425		0.6490		0.7001	

**Table 2:** Top 5 positive highest-weighted relations and top 5 negative highest-weighted relations.

Scenarios and relations			Scenario 1			Scenario 2			Scenario 3	
**Top 5 negative highest-weighted relations**										
	1	Neoplasms of unspecified nature or uncertain behavior		Consultation, evaluation, and preventative care	Consultation, evaluation, and preventative care		Dihydropyridines	Quinolone antibiotics		Suture of skin and subcutaneous tissue
	2	Neoplasms of unspecified nature or uncertain behavior		Lens and cataract procedures	Consultation, evaluation, and preventative care		Diseases of white blood cells	Quinolone antibiotics		Lens and cataract procedures
	3	Neoplasms of unspecified nature or uncertain behavior		Hyperlipidemia	Consultation, evaluation, and preventative care		Upper gastrointestinal endoscopy, biopsy	Quinolone antibiotics		Essential hypertension
	4	Neoplasms of unspecified nature or uncertain behavior		Diabetes mellitus with complications	Consultation, evaluation, and preventative care		Other CT scan	Quinolone antibiotics		Diagnostic ultrasound of head and neck
	5	Neoplasms of unspecified nature or uncertain behavior		Diagnostic ultrasound of head and neck	Diseases of white blood cells		Dihydropyridines	Quinolone antibiotics		Psychological and psychiatric evaluation and therapy
**Top 5 positive highest-weighted relations**										
	1	Routine chest x-ray		Electrocardiogram	Substance-related disorders		Electrocardiogram	Schizophrenia and other psychotic disorder		Substance-related disorders
	2	Routine chest x-ray		Other laboratory	Substance-related disorders		Other laboratory	Schizophrenia and other psychotic disorder		Diagnostic procedures on nose, mouth, and pharynx
	3	Routine chest x-ray		Heart valve disorders	Substance-related disorders		Routine chest x-ray	Diagnostic ultrasound of head and neck		Arthrocentesis
	4	Electrocardiogram		Other laboratory	Electrocardiogram		Inguinal and femoral hernia repair	Diagnostic ultrasound of head and neck		Substance-related disorders
	5	Electrocardiogram		Heart valve disorders	Substance-related disorders		Coronary atherosclerosis and other heart disease	Substance-related disorders		Other diagnostic radiology and related techniques

## Discussion

### Principal Findings

Based on this study’s results, we found that some potential candidates might be relevant to ADRD risk prediction and treatment. Our self-explainable GNN prediction method reveals the underneath connections between medical codes for ADRD risk prediction. Some code pairs have been shown to accelerate ADRD progression, while others exhibit potential to slow down its development. When implementing our relation importance interpretation method, the GNN results are explainable, setting it apart from other deep learning models. Moreover, several code pairs extracted from the GNN align with findings from previous research. Those code pairs that are not proven could offer valuable insights beyond the scope of current studies, opening up avenues for further investigation and enhancing our understanding of ADRD risk prediction. Table 2 shows the top 5 positive highest-weighted relations and the top 5 negative highest-weighted relations. In the following sections, we will present examples of code pairs derived from the GNN model results and highlight their significance based on validated evidence from previous studies.

This study found that certain pairs of medical codes can be associated with a decreased likelihood of an ADRD diagnosis. For instance, the treatment of more acute conditions, such as cancer or neoplasms, may delay the diagnosis of ADRD. We hypothesize that “neoplasms of unspecified nature or uncertain behavior” may be correlated with higher health care use or more frequent physician visits, similar to the code “consultation, evaluation, and preventative care.” The cooccurrence of these 2 types of coding could potentially lower the risk of ADRD. Regular health care visits could potentially reduce the risk of ADRD by improving modifiable risk factors and mitigating social isolation in older patients. Lee et al [32] revealed that cataract extraction is linked to a reduced risk of developing dementia among older adults. Cataract extraction has been associated with enhanced engagement in intellectually stimulating activities, such as reading and video consumption, as well as increased physical activity. These changes in lifestyle and cognitive engagement following cataract surgery may contribute to a delay in the onset of ADRD. Consequently, the second node pair involving “neoplasms of unspecified nature or uncertain behavior” and “lens and cataract procedures” also holds relevance and supports the observed association. In scenario 2, Peters et al [33] have indicated that the use of calcium channel blockers, specifically dihydropyridines, is associated with a lower decline in cognitive function compared to other hypertensive treatments. Thus, the presence of the “consultation, evaluation, and preventative care” and “dihydropyridines” nodes pair ranking first in importance is consistent with the reported associations. The most frequently appearing node in scenario 3 is “quinolone antibiotics.” According to the study by Pham et al [34], it is a class of medication commonly prescribed to treat various bacterial infections and is primarily used for its antimicrobial properties [34]. Additionally from a study by Gao et al [35], their review study indicates that the brain inflammation caused by microbial infections may be one of the etiologies of ADRD, and antibiotics as novel treatments may be beneficial for delaying the development of ADRD. Quinolones exhibit a distinct pharmacokinetic profile characterized by a higher cerebrospinal fluid to serum concentration ratio compared to other commonly prescribed antibiotics [36]. This unique attribute may underlie the observed robust negative correlation between quinolone administration and the development of ADRD, distinguishing its potential protective effect from that of other antibiotics. The use of quinolones likely correlates with younger age, as its use in older adults is less frequent due to the increased risk of tendon rupture. However, this is less likely to explain its negative correlation with the onset of ADRD in our age-matched cohorts. So, in other words, it can be hypothesized that “quinolone antibiotics” may potentially exhibit a slowing effect on the progression of ADRD. Combined with the aforementioned node “lens and cataract procedures,” the observed association of this node pair holds validity and is worth further investigation.

This study also found certain medical codes to be positively associated with a higher likelihood of an ADRD diagnosis. This can be explained by the fact that Alzheimer disease, to a certain degree, is a “diagnosis of exclusion.” Procedures like “routine chest x-ray” and “electrocardiogram” are commonly used as initial steps in diagnosing altered mental status, which is often the first sign of ADRD. A chest x-ray is often used to rule out any underlying pneumonia, while an electrocardiogram may be used to rule out arrhythmia [37]. Similarly, “diagnostic ultrasound of head and neck” is commonly done to rule out conditions like carotid artery clot, stenosis, or plaque in the setting of stroke workups. Once patients begin to verify these initial diagnoses of altered mental status, they are more likely to undergo comprehensive and relevant testing to exclude other potential causes of the symptoms, which may potentially lead to a timely determination of ADRD. Several studies have also found that alcohol and drug use could affect mental state and cognitive function [38]. People who abuse intoxicating substances for a considerable period may develop dementia or accelerate the neurological damage associated with Alzheimer [39].

From the modeling aspect, to the best of our knowledge, our approach offers distinct advantages in comparison to previous studies on the early diagnosis of ADRD with or without GNN methods. For instance, Li et al [40] used a gradient boost tree and logistic regression to assess ADRD risk using EHR data from the OneFlorida+ Clinical Research Consortium. They identified significant clinical and social factors through SHAP values; however, these factors were commonly known risk factors. In contrast, our findings unveil potential risk factors and explain the interaction among these factors in ADRD prediction. While VGNN demonstrates good interpretability by showcasing attention weights among features, it fails to explain how these features positively or negatively impact ADRD prediction [23]. On the other hand, our model offers interpretations of potential risk factors and illustrates their influence on outcomes. Furthermore, our proposed self-explainable framework mitigates the potential bias resulting from the prevalence of medical codes. Klepl et al [24] conducted electroencephalography-based ADRD prediction using GNN methods. As medical image data are unavailable for every patient during routine examinations, limitations arise due to the restricted user cases. Furthermore, they only assessed model performance against baseline models without providing any feature interpretation. Conversely, our method enhances interpretability by leveraging more accessible data, thereby promoting broader applicability and understanding in ADRD prediction. Overall, this is the first work that proposes a self-explainable framework, providing a feature-important explanation in the context of ADRD risk prediction leveraging relational information within a graph. Compared with other studies on ADRD risk predictions, our method can directly interpret the relationship’s importance within the training process. It does not require any additional post hoc explanation methods, such as GNNExplainer [26]. In other words, within our framework, it takes no additional time to get an explanation.

In summary, we showed that using the GNN approach for ADRD prediction has better performance compared to baseline models. Moreover, with the incorporation of our relation importance method, the model’s results become explainable, providing valuable insights into the underlying factors contributing to ADRD risk prediction.

### Limitations

Our prediction does not incorporate time information into the modeling process. In this study, we aggregated 3 years of records into a single representation and treated them equally without considering their temporal sequence. In the real-world clinical setting, medical events, procedures, or medications obtained at different times should carry different levels of significance. In other words, events occurring closer in time to the prediction window are expected to have a greater impact on the disease prediction. In our future study, we could use a time series model and positional encoding method to establish connections between patients’ multiple visit records for more accurate predictions and provide more valuable insights into ADRD prediction.

On the other hand, it is important to note that certain predicted correlations may not causally assist clinicians in diagnosing ADRD. For instance, initiating tests for early detection of altered mental status might lead patients to identify ADRD through various related tests. Nonetheless, from the clinician’s perspective, ordering these test results may not be helpful for early ADRD prediction. In our future work, we could try to exclude these “subjective patient-related factors” and instead focus on investigating more objective risk factors that could potentially influence the prediction of ADRD.

### Conclusion

In this study, we used an advanced self-explainable GNN approach and developed a relation importance interpretation method for the ADRD risk prediction task based on claims data sources. The VGNN model’s effectiveness was evaluated across 3 distinct scenarios, with comparisons made against RF and LGBM ML models. The model’s performance achieved satisfactory results. In addition, we provided the interpretation for the node pairs extracted from the KG, which was generated from the VGNN model. Furthermore, we demonstrated the results’ future applicability and explained the important node pairs that align with previous research findings. This work contributes to the advancement of ADRD prediction models and reinforces the importance of interpretable results for informed clinical decision-making and early detection, etc.

## Appendix

Multimedia Appendix 1 The model performance (area under the receiver operating characteristic scores) for Alzheimer disease and related dementia risk prediction. Matched cohort: employ 1:1 propensity score match for case and control in the original training data (i.e., 80% of full data), to achieve a balanced dataset and train the models, and test the models in the hold-out 20% of full data. Subset cohort: use around 10% of the Matched cohort (i.e., 20,000 for both case and control) to train the models, and test the models in the hold-out 20% of full data.

## Abbreviations

ADRD: Alzheimer disease and related dementias
AUROC: area under the receiver operating characteristic curve
DeepDDS: Deep Learning for Drug-Drug Synergy prediction
EHR: electronic health record
GAT: graph attention network
GCT: graph convolution transformer
GNN: graph neural network
ICD-9: International Classification of Diseases, 9th revision
ICD-10: International Classification of Diseases, 10th revision
LGBM: light gradient boost machine
ML: machine learning
RF: random forest
VGNN: Variationally regularized encoder-Decoder Graph Neural Network
