# Supplementary figures and images for: Self-Explainable Graph Neural Network for Alzheimer Disease and Related Dementias Risk Prediction: Algorithm Development and Validation Study
Source: JMIR Aging. 2024 Jul 8;7:e54748. doi: 10.2196/54748 (PMC11263893; doi:10.2196/54748)

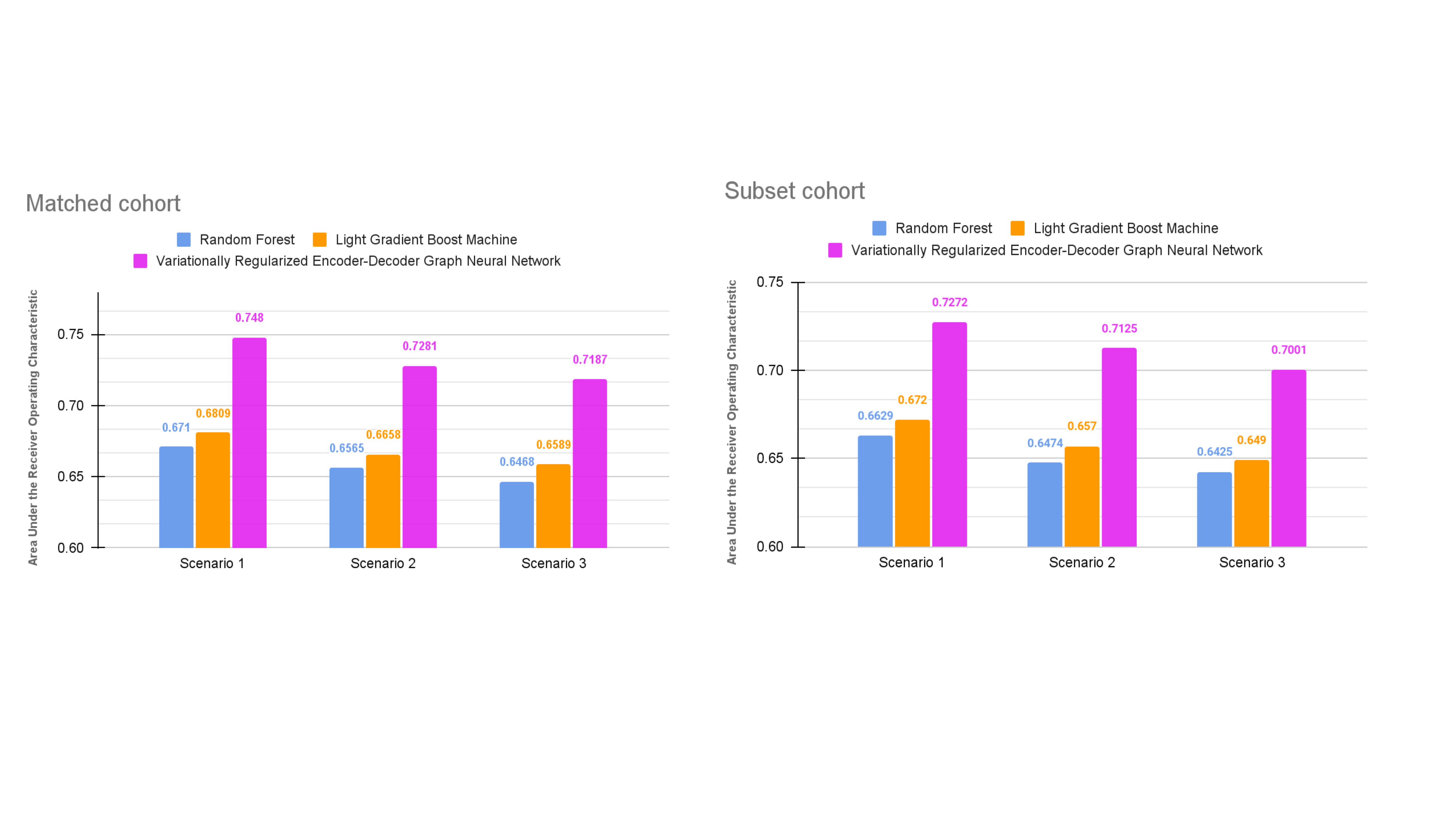

Supplement: Multimedia Appendix 1 [file aging_v7i1e54748_app1.png]
